# MRI-based habitat analysis of vascular and nerve invasion in the tumor microenvironment: an advanced approach for prostate cancer diagnosis

**DOI:** 10.3389/fonc.2025.1541413

**Published:** 2025-04-17

**Authors:** Bo Guan, Cong Huang, Yalei Wang, Jialong Zhang, Xiaowei Li, Zongyao Hao

**Affiliations:** ^1^ Department of Urology, Fuyang People’s Hospital of Anhui Medical University, Fuyang, China; ^2^ Department of Urology, the First Affiliated Hospital of Anhui Medical University, Hefei, China; ^3^ Department of Radiology, Fuyang People’s Hospital of Anhui Medical University, Fuyang, China; ^4^ Department of Nephrology, Fuyang People’s Hospital of Anhui Medical University, Fuyang, China; ^5^ Institute of Urology, Anhui Medical University, Anhui Province Key Laboratory of Genitourinary Diseases, Anhui Medical University, Hefei, China

**Keywords:** prostate cancer, radiomediography, habitat imaging, tumor microenvironments, MRI

## Abstract

**Purpose:**

This study aims to detect vascular and neural invasion in prostate cancer through MRI, utilize habitat analysis of the tumor microenvironment, construct a radiomic feature model, thereby enhancing diagnostic accuracy and prognostic assessment for prostate cancer, ultimately improving patients’ quality of life.

**Methods:**

We retrospectively collected records of 400 patients with pathologically verified prostate cancer from January to December 2023. We developed a radiomic features model within the tumor habitat using MRI data and identified independent risk factors through multivariate analysis to construct a clinical model. Finally, we assessed the performance of these features using the DeLong test (through the area under the receiver operating characteristic curve, AUC), evaluated the calibration curve with the Hosmer-Lemeshow test, and performed decision curve analysis.

**Results:**

In the training set, the optimal algorithm based on the intratumoral heterogeneity score had an AUC value of 0.882 (CI: 0.843-0.921); in the test set, the AUC value was 0.860 (CI: 0.792-0.928). The traditional radiomics model (considering the entire tumor) had an AUC value of 0.761 (CI: 0.695-0.827) in the training set and 0.732 (CI: 0.630-0.834) in the test set. The combined model that integrates habitat scores and Gleason scores had an AUC value of 0.889 (CI: 0.8509-0.9276) in the training set and 0.886 (CI: 0.8183-0.9533) in the test set, outperforming the single models.

**Conclusions:**

By deeply analyzing the tumor microenvironment and combining radiomics models, the diagnostic precision and predictive accuracy of vascular and nerve invasion in prostate cancer can be significantly improved. This approach provides a valuable tool for optimizing treatment plans, improving patient prognosis, and reducing unnecessary medical interventions.

## Introduction

Prostate cancer (PCa) prevalence is on the rise, second only to lung cancer, making it the most common form of male-specific malignancy and a major contributor to fatalities due to cancer (1–4) (5). Perineural invasion (PNI) is highly prevalent in PCa, observed in up to 75% of surgical resection specimens (6). PNI is an ominous clinical and pathological characteristic of PCa, which has been associated with cancer pain, adverse pathological features, elevated biochemical recurrence rates, increased risk for bone metastasis and diminished overall survival (7–9), It is defined as the invasion of cancer cells into the nerves, around the nerves, and through the nerves, and is an indicator of low prognosis and survival rate in PCa (10). While organ-confined PCa can be effectively managed, the metastatic disease originating from the extracapsular extension is mostly incurable (11). Interestingly, over 50% of surgical specimens have extracystic dilation as a result of PCa that spreads mainly or completely in the surrounding space of the nerves (12). Currently, there is no relevant test to analyze the prognosis of prostate cancer patients, and clinicians are limited to biopsies.

The task of diagnosing and predicting the progression of diseases with imaging techniques, especially tumors, is challenging due to the complexity of the biological processes involved. Prognosis can be improved by aAccurate diagnosis and prediction of tumor lethality which are essential for optimizing treatment options. However, traditional diagnostic tools often lead to subjective interpretations due to limited information, resulting in inaccurate decisions. For example, clinicians often draw blood in patients with prostate tumors, for assessing the prostate-specific antigen (PSA) for diagnosing early prostate cancer, but elevated PSA levels may be caused by multiple factors, which may result in overdiagnosis and over-treatment (13–16). To address these limitations, we have introduced a novel approach that utilizes advanced medical image analysis techniques to improve diagnostic accuracy.

The limitations in the diagnosis and treatment of prostate cancer, including the issues of overdiagnosis and overtreatment, are particularly significant in countries like China, where the incidence of prostate cancer is continuously rising. This study proposes an innovative method by deeply analyzing the tumor microenvironment to capture its heterogeneity and complexity, which not only improves diagnostic accuracy but also significantly enhances predictive precision. The application of MRI imaging technology allows for a comprehensive examination of different tumor regions, identifying patterns and correlations that may have been missed by traditional methods.

Moreover, this study constructs a radiomics model that combines feature selection, detailed analysis of the tumor microenvironment, and assessment of clinical relevance. This comprehensive approach helps identify the most informative and prognostic imaging features. By integrating these features with an in-depth analysis of the tumor microenvironment, a comprehensive tumor atlas is created, capturing the biological and clinical characteristics of the tumor. This atlas provides a valuable basis for optimizing treatment plans, improving patient prognosis, and reducing unnecessary medical interventions.

For Chinese patients, the significance of this research is particularly pronounced. With the incidence of prostate cancer on the rise among Chinese men, often accompanied by higher mortality rates and poor prognosis, this method not only enhances diagnostic efficiency but also offers a scientific basis for personalized treatment plans. This can help improve the overall survival rate and quality of life for Chinese patients with prostate cancer.

In this study, we screened 400 patients with PCa through a series of rigorous inclusion and exclusion criteria. These patients were randomized to two groups, namely the training group and the testing group, to ensure the effectiveness and reliability of the model.

## Methods

### Patient selection

Records of 400 patients with prostate cancer cases from January 2023 to December 2023 were collected in June 2024. The inclusion criteria were: (1) Patients above 18 years of age; (2) Patients must have had a definitive pathological diagnosis of prostate cancer confirmed through prostate biopsy or radical surgery. This criterion ensured that only confirmed cases of prostate cancer were included in the study.; (3) All patients’ medical records had to be comprehensive, including all relevant clinical variables and radiomics histological data. This ensured that sufficient data was available for analysis and modeling. (4) Patients were required to be conscious and motionless during the imaging investigations to eliminate errors caused by passive positions or motion artifacts due to unclear consciousness. This criterion ensured the quality and reliability of the MRI images used in the study. (5) Patients who had not received endocrine therapy prior to the MRI examination were included to avoid potential confounding factors that may arise from therapy-induced changes in the prostate. (6) There were no specific restrictions on the time interval between the MRI examination and surgery. However, all relevant clinical data and imaging findings from the MRI examination had to be available and recorded accurately. Exclusion criteria were: (1) Histologically undiagnosed prostate cancer; (2) Patients with severe comorbidities, such as cardiovascular or circulatory system diseases, were excluded to avoid potential confounding factors that may affect the accuracy of the MRI imaging and subsequent radiomics analysis. This criterion ensured that the study population was relatively homogeneous in terms of health status, allowing for more reliable and valid analysis results. The workflow of radiomics analysis involves dividing the samples into two groups ina 2:1 ratio: a training group containing 270 samples and an internal testing group containing 130 samples.

### Workflow of radiomics analysis

**Figure 1:**
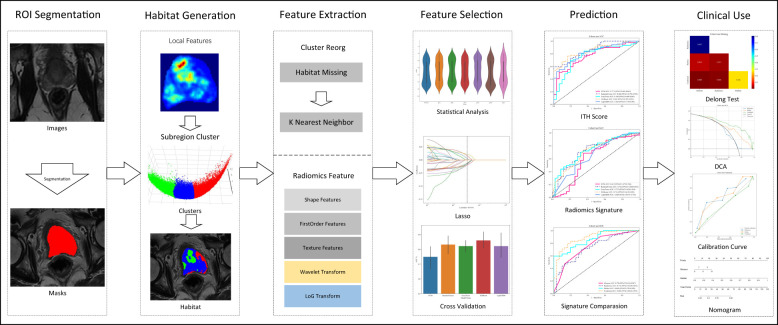
Overall workflow of this study.

In this study, a novel approach was introduced that aimed to address the complex challenges inherent in medical image analysis, with a focus on enhancing diagnostic accuracy through two key advancements. Firstly, an in-depth analysis of the tumor microenvironment is performed by comprehensively examining different tumor zones; this has improved our predictive accuracy significantly resulting in informed clinical decision-making. Secondly, the advanced radiomics model leverages an combined approach merging feature selection, detailed analysis of the tumor microenvironment, and assessment of clinical significance, thereby enhancing the accuracy of tumor lethality predictions. **Figure 1** illustrates the detailed workflow of our methodology, demonstrating its potential to evidently improve prognostic capabilities in the field.

### Image acquisition

All patient images were taken using a high-resolution 3.0 Tesla Philips Magnetic Resonance Imaging machine. This state-of-the-art imaging system was used to guarantee that the highest quality of diagnostic images were recordeed. The MR750 WIDE MRI machine is renowned for its wide field of view, which allows for visualizing larger body parts and improved capturing the images of anatomical structures. Additionally, its advanced gradient system and high bandwidth capabilities enable the acquisition of high-resolution, detailed images promptly (**Supplementary Table S1**).

### Image segmentation

The demarcation of the Region of Interest (ROI) was conducted using ITK-SNAP by two individual experienced radiologists, working independently. In cases of disagreement, the opinion of a third expert radiologist, possessing two decades of professional experience was sought to make the final determination, thereby guaranteeing the precision and dependability of the ROI identification.

### Data preprocessing

In our study, voxel spacing was standardized to 1mm×1mm×1mm across all analyzed image volumes using a fixed resolution resampling technique (17). Moreover, we normalized the MR value to a range of -120 to 180. This resulted in precise image comparisons and significantly enhanced the accuracy and reliability of our analytical results.

### Intratumor heterogeneity analysis

#### Subregion generation

**Figure 2:**
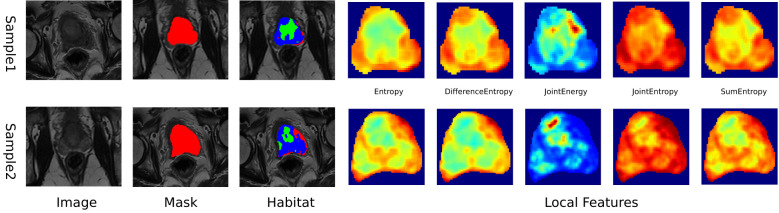
Schematic diagram of intratumoral heterogeneity region segmentation.

In this study, MRIs were used to extract local features, including entropy and energy, from each voxel within the Volume of Interest (VOI), using a 3x3x3 moving window technique, resulting in a 19-dimensional feature vector for each voxel, as outlined in **Supplementary 1A**. K-means clustering algorithm was used to segment the VOI into distinct subregions, with the number of clusters ranging from 3 to 10, with the optimal number of clusters determined by the Calinski-Harabasz (CH) score (18),. This approach provided a detailed characterization of intratumor heterogeneity, enhancing our understanding of the tumor’s structural complexity (**Figure 2**). Further details of our methodology and the implications of these findings are discussed in **Supplementary 1A**.

#### Feature extraction

Each segmented subregion, including the whole intra tumor VOI, a comprehensive classification of handcrafted radiomic features was performed into three main groups: geometric, intensity, and texture. Geometric features measure the tumor’s shape and spatial extents, intensity features assess the brightness levels of voxels, and texture features analyze spatial patterns within the tumor using advanced techniques such as the Gray Level Co-occurrence Matrix (GLCM), Gray Level Run Length Matrix (GLRLM), Gray Level Size Zone Matrix (GLSZM), and Neighborhood Gray Tone Difference Matrix (NGTDM).

Radiomic features were extracted from the entire VOI to perform intra-tumor analysis including specific subregions identified within the tumor. K-Nearest Neighbors (KNN) method was used to ensure consistent labeling across different habitat regions in order to manage the unclustered areas resulting from the unsupervised nature of our clustering algorithm, we employed the. Feature extraction was executed using the pyradiomics tool (version 3.0.1), which adheres to the strict standards set by the Imaging Biomarker Standardization Initiative (IBSI).

A pre-fusion technique was used to merge the features from the respective subregions, thereby creating a robust and informative combined feature set. This approach significantly enhances the predictive efficacy of our models in assessing treatment efficacy and tracking changes in tumor characteristics.

#### Feature selection

Intraclass Correlation Coefficient (ICC) was used to ensure the reliability of radiomic features, selectively retaining features that exhibited an ICC > 0.8 as evaluated by two independent physicians. This strategy guaranteed that only features demonstrating high consistency were subsequently analyzed. We then normalized the feature distribution according to the mean and standard deviation of the training cohort. Statistical evaluation involved t-tests, and the significance threshold set at p<0.05, to only retain features demonstrating statistical significance.

For correlation analysis, we utilized Pearson’s correlation to identify and eliminate highly correlated features, setting a cutoff threshold at 0.9. Minimum Redundancy Maximum Relevance (mRMR) algorithm was sued to further refine the analysis, which optimized our feature set to 32 by effectively balancing relevance and redundancy.

Least Absolute Shrinkage and Selection Operator (LASSO) regression was used to further improve the selection process for our radiomic signature. This method simplifies the model by penalizing the regression coefficients, effectively removing inappropriate features. The optimal regularization parameter λ was identified through 10-fold cross-testing, ensuring the selection of the most predictive features. This comprehensive methodology, using ICC filtering and LASSO regression, solidifies a predictive and robust radiomic signature.

#### Signature building

Machine learning models were developed using features selected via LASSO to predict the Radiomics Signature and Intratumor Heterogeneity (ITH) score. Furthermore, Grid-Search algorithm was used to optimise the models’ hyperparameters across 5-fold cross-testing.

##### Radiomics signature (Radiomics)

The refined features were used combined to develop advanced algorithms, employing Logistic Regression (LR) for linear modeling and Random Forest for handling complex structures. This approach enabled the formation of a nuanced risk model that effectively captured the intricacies within the data.

##### Clinical signature

Multiple Instance Learning (MIL) approach was used to ensure uniformity across our analysis by including all clinical features in the model by the same machine learning algorithms.

##### Combined model

We conducted univariate and stepwise multivariate analyses of the selected clinical features in the model to validate the efficacy of our integrated model. Only significant (p<5) features were merged with the MIL features to form the final combined model. This rigorous approach ensured comprehensive evaluation and integration of significant clinical predictors, improving the overall predictive capability of our model.

### Statistical analysis

Shapiro-Wilk test was use to evaluate the normality of clinical features. T-test or the Mann-Whitney U test was used to analyze the continuous variables, based on their distribution. Chi-square (χ²) tests were used to assess the categorical variables. The baseline characteristics of all cohorts are detailed in **Table 1**. P-values between different cohorts exceeded 0.05, indicating no significant differences and confirming unbiased group allocation.

**Table 1:** Baseline characteristics of the two (training and test) cohorts.

Feature_name	ALL	Train	Test	p-value
PSA	52.40 ± 154.97	44.08 ± 108.46	71.81 ± 228.91	0.458926607
Age	70.36 ± 7.39	70.05 ± 7.19	71.08 ± 7.82	0.107417592
Gleason				0.934392706
6	66 (16.50)	44 (15.71)	22 (18.33)	
7	181 (45.25)	129 (46.07)	52 (43.33)	
8	81 (20.25)	55 (19.64)	26 (21.67)	
9	65 (16.25)	47 (16.79)	18 (15.00)	
10	7 (1.75)	5 (1.79)	2 (1.67)	

OnekeyAI platform, version 3.5.12, using Python 3.7.12 was utilized to perform all statistical analyses. Statistical computations were executed using Statsmodels version 0.13.2. PyRadiomics version 3.0.1was used to conduct the Radiomics feature extraction. Machine learning algorithms, including the Support Vector Machine (SVM), were implemented using Scikit-learn version 1.0.2.

## Results

### Clinical features

A rigorous univariate analysis was conducted encompassing all clinical features, primarily focusing on the calculation of odds ratio (OR) and its corresponding p-values for each attribute. Moreover, the Gleason score exhibited p-values less than 0.05, thus reflecting statistical significance. Due to this significance, Gleason was subsequently chosen as a crucial clinical comparison factor for our subsequent analyses (**Table 2**; **Supplementary Figures S2A, B**).

**Table 2:** Univariable and multivariable analysis of clinical features.

feature_name	OR	OR lower 95%CI	OR upper 95%CI	p_value	OR	OR lower 95%CI	OR upper 95%CI	p_value
Age	0.999	0.993	1.005	0.804				
PSA	1.000	1.000	1.001	0.342				
Gleason	1.099	1.055	1.145	<0.05	1.099	1.055	1.145	<0.05

The incidence of prostate disease increases as the age increases, year by year, and similar trends are seen with the incidence of prostate tumors (19). PSA levels are used in the diagnosis of prostate cancer, with clinicians often using PSA as a tumor marker (20), because PSA is a specific indicator of prostate tumors (21). Furthermore, the Gleason score has been associated with course of the disease and prognosis with certain advantages in predicting staging indicators such as tumor extent, lymph node metastasis, and distant metastasis. The Gleason score is also used for predicting the effect of tumor treatment (surgical and hormonal treatment). However, the changes in the blood PSA levels is easily affected by various factors, such as other prostate diseases, and several different surgical operations performed on the prostate, Even examinations on the prostate such as DRE can cause the PSA levels to increase in the blood PSA level even if there is only a slight injury. Thus, PSA does not accurately predict the occurrence of prostate tumors. Therefore, the need of the hour is to develop methods to accurately predict the presence of prostate tumors at an early stage, metastasis and their prognosis including preventive measures. This will result in a better quality of life for the patients with prostate cancer and their overall survival.

### Handcrafted features

We assessed the impact of varying the number of clustering centers from 3 to 10 on the efficacy of our analysis (**Supplementary Figure S3A, B**).

**Figure 3:**
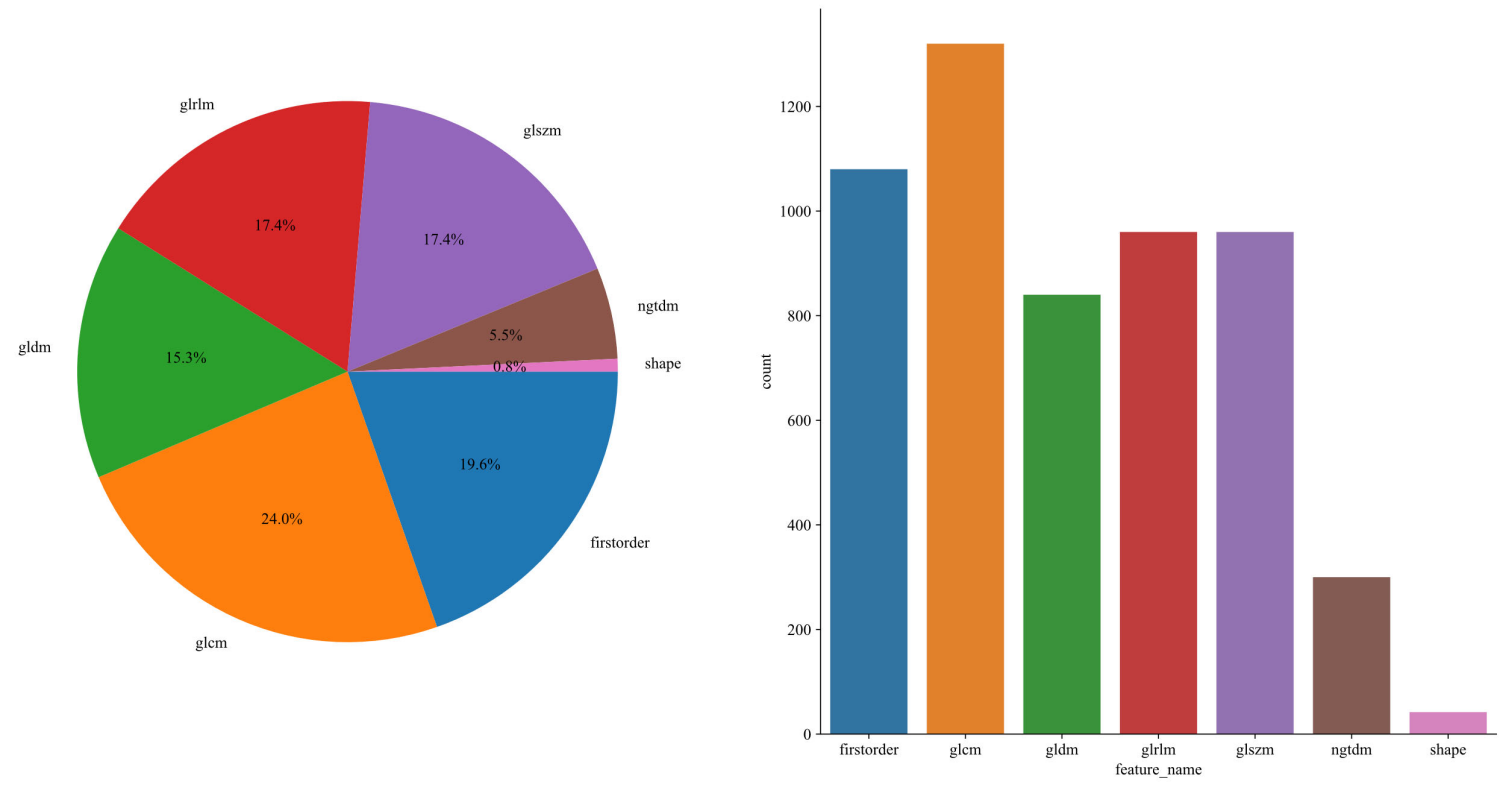
The number and proportion of manually extracted features.

1,834 unique radiomic features were extracted in this study, including shape, first-order, and texture categories. 360 first-order and 14 shape features, and a variety of texture features were identified. The final ITH score included features from three subregions, totaling 5,502 features (**Figure 3**).

**Figure 4:**
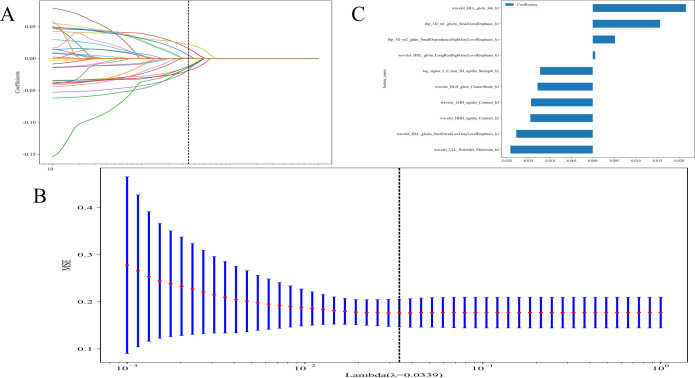
**(A)** Coefficients of 10-fold cross validation; **(B)** The mean standard error (MSE) of 10-fold cross validation; and **(C)** The histogram of the Rad-score based on the selected features.

LASSO technique was used for feature extraction. LASSO logistic regression model was used to detect significant non-zero coefficients for the Rad-score. The depiction of these coefficients, along with the mean standard error (MSE) calculated through a 10-fold cross-testing technique, is exhibited in **Figure 4**. Radiomic features, are presented in **Supplementary 2A**.

### Radiomics results

We evaluated the efficacy of models that only targeted intra-tumoral regions against those that incorporated segmentation specific to Intratumoral Heterogeneity Regions (ITHRs) within the tumor. Our findings revealed that models leveraging the Intratumoral Heterogeneity (ITH) score demonstrated a marked superiority over conventional models that encompassed the entire tumor.

#### Intratumor heterogeneity score

Attaining an AUC of 0.882 in the training cohort, with a confidence interval spanning from 0.843 to 0.921, and a corresponding AUC of 0.860 in the testing cohort, falling within the range of 0.792 to 0.928, the RandomForest model demonstrated a consistently high proficiency in distinguishing between the groups. This characteristic is particularly remarkable when juxtaposed with other models, such as SVM, ExtraTrees, XGBoost, and LightGBM.

**Figure 5:**
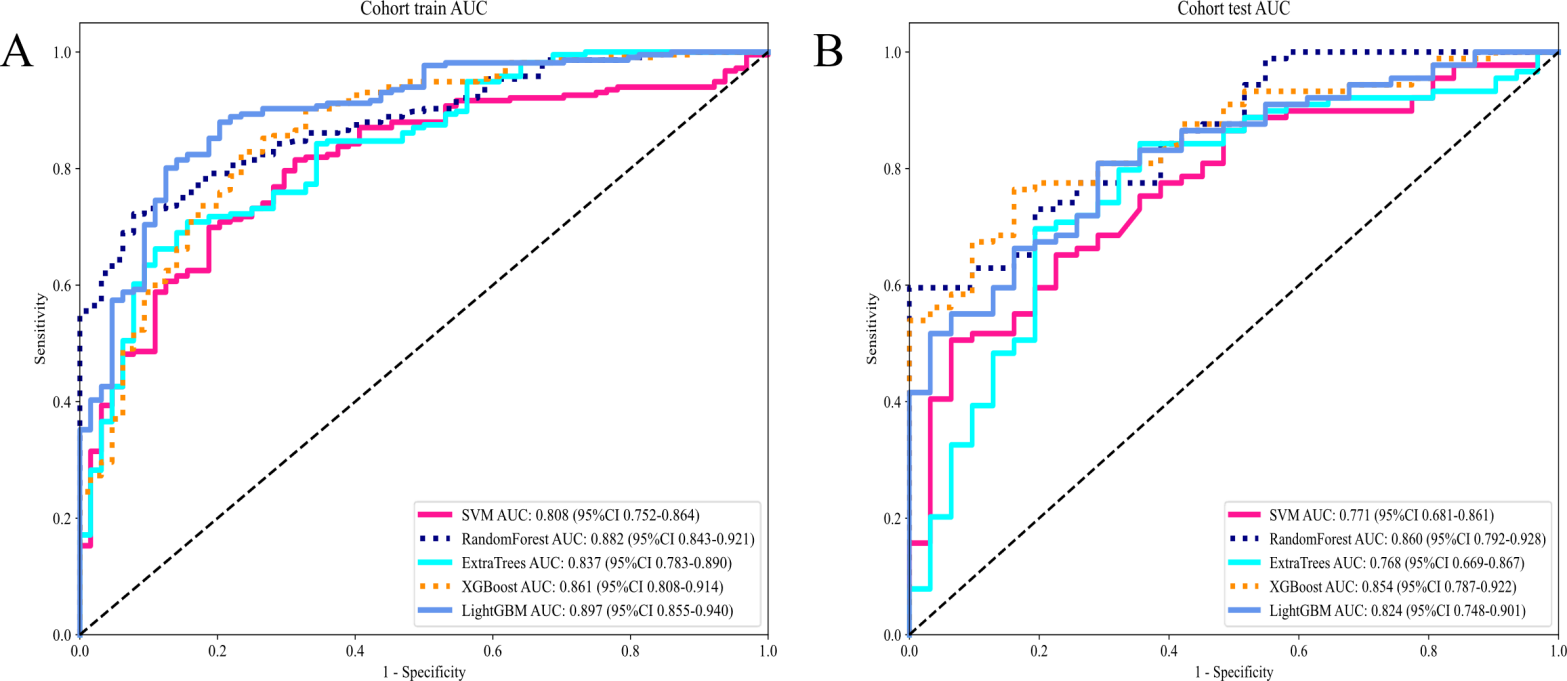
**(A)** Receiver operating characteristic (ROC) curves of different models in train cohort of Intratumor Heterogeneity; **(B)** ROC Curves of Different Models in testing cohort of Intratumor Heterogeneity.

The AUC demonstrates the RandomForest model’s effectiveness in executing the categorization (test and training) task in this specific domain. Among the models assessed, RandomForest was the most outstanding model which was validated by its superior performance in both training and testing phases indicating robust generalizability and a dependable predictive capability. This underscores its aptness for deployment in analogous scenarios where predictive accuracy is paramount (**Table 3** **) (** **Figures 5A, B**).

**Table 3:** Model performance of different machine learning algorithms in each cohort.

Model_name	Accuracy	AUC	95% CI	Sensitivity	Specificity	PPV	NPV
SVM	0.707	0.808	0.752 - 0.864	0.676	0.812	0.924	0.426
SVM	0.708	0.771	0.681 - 0.862	0.719	0.677	0.865	0.457
Random Forest	0.764	0.882	0.843 - 0.921	0.736	0.859	0.946	0.491
Random Forest	0.758	0.860	0.792 - 0.928	0.764	0.742	0.895	0.523
Extra Trees	0.707	0.837	0.783 - 0.890	0.662	0.859	0.941	0.430
Extra Trees	0.717	0.768	0.669 - 0.867	0.697	0.774	0.899	0.471
XGBoost	0.768	0.861	0.808 - 0.914	0.759	0.797	0.927	0.495
XGBoost	0.767	0.854	0.787 - 0.922	0.775	0.742	0.896	0.535
LightGBM	0.818	0.897	0.855 - 0.940	0.806	0.859	0.951	0.567
LightGBM	0.775	0.824	0.748 - 0.901	0.798	0.710	0.887	0.550

#### Radiomics signature

The evaluation of the AUC across various models reveals a spectrum of performances, with the ExtraTrees model standing out as the preeminent one with the highest AUC values. The ExtraTrees model attained an AUC of 0.761(CI: 0.695 to 0.827) for the training group while for the testing cohort, it achieved an AUC of 0.732 (CI: 0.630 to 0.834). This remarkable performance signifies the robust ability of the ExtraTrees model to distinguish between different classes, surpassing the performances of other models, such as SVM, RandomForest, XGBoost, and LightGBM, particularly during the training phase (**Table 4**).

**Table 4:** Model performance of different machine learning algorithms in each cohort.

Model_name	Accuracy	AUC	95% Confidence interval (CI)	Sensitivity	Specificity	PPV	NPV
SVM	0.736	0.615	0.531 - 0.698	0.856	0.328	0.811	0.404
SVM	0.733	0.613	0.479 - 0.746	0.933	0.161	0.761	0.455
RandomForest	0.629	0.732	0.662 - 0.801	0.606	0.703	0.873	0.346
RandomForest	0.717	0.726	0.620 - 0.832	0.753	0.613	0.848	0.463
ExtraTrees	0.732	0.761	0.695 - 0.827	0.759	0.641	0.877	0.441
ExtraTrees	0.742	0.732	0.630 - 0.834	0.843	0.452	0.815	0.500
XGBoost	0.625	0.720	0.652 - 0.787	0.602	0.703	0.872	0.344
XGBoost	0.717	0.716	0.612 - 0.820	0.753	0.613	0.848	0.463
LightGBM	0.668	0.728	0.662 - 0.795	0.671	0.656	0.868	0.372
LightGBM	0.692	0.660	0.543 - 0.776	0.753	0.516	0.817	0.421

**Figure 6:**
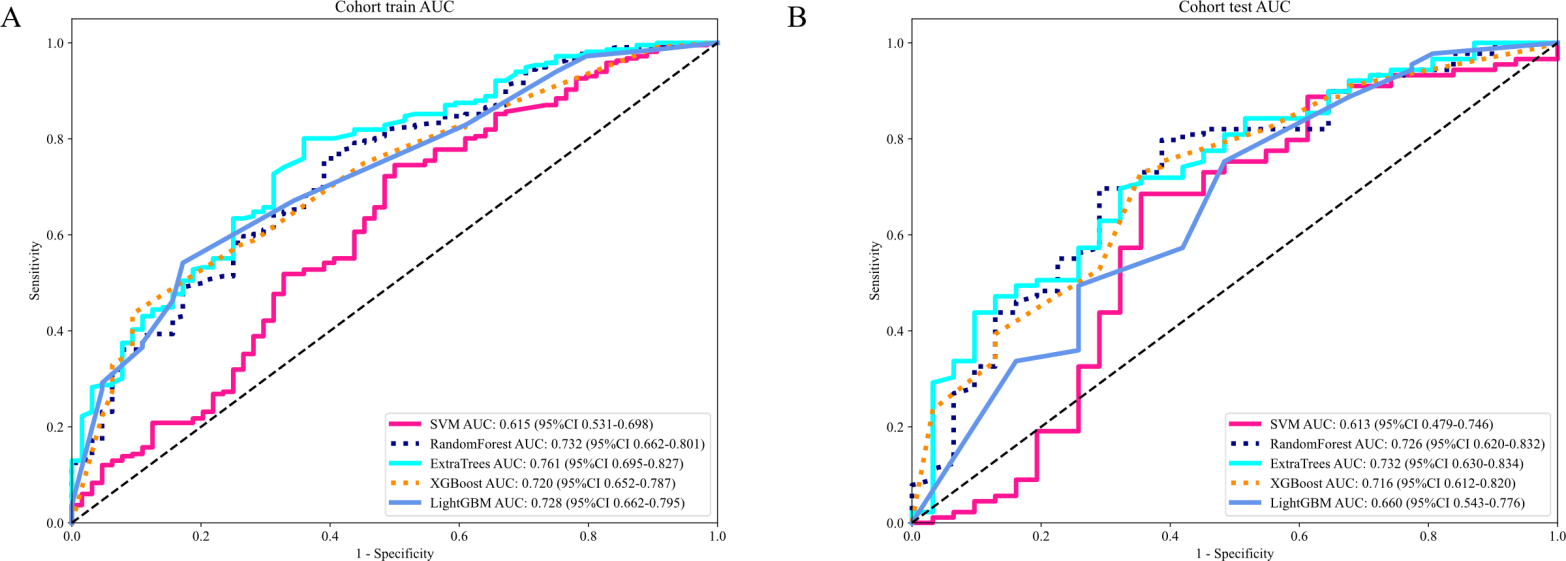
**(A)** Receiver operating characteristic (ROC) curves of different models in train cohort of Radiomics signature; **(B)** ROC Curves of Different Models in testing cohort of Radiomics signature.

This consistency not only underscores the Extra Tree model’s excellent generalization ability but also renders it a favored choice for submissions that necessitate dependable classification presentation (**Figure 6**).

#### Signature comparation

The evaluation of AUC values across diverse signatures reveals that the combined model, encompassing Habitat and Gleason scores, surpasses individual models in discriminative prowess. The combined model attained an AUC of 0.889 (CI: 0.8509 to 0.9276) in the training group reflecting outstanding classification efficiency. Similarly, in the testing cohort, it reached an AUC of 0.886(CI: 0.8183 to 0.9533). This performance surpasses that of individual Gleason, Radiomics, and Habitat models in both cohorts (**Table 5**).

**Table 5:** Prediction performance of intratumor heterogeneity region based rad signatures.

Signature	Accuracy	AUC	95% CI	Sensitivity	Specificity	PPV	NPV	Cohort
Gleason	0.771	0.648	0.5757 - 0.7200					Train
Radiomics	0.732	0.761	0.6952 - 0.8266	0.759	0.641	0.877	0.441	Train
Habitat	0.764	0.882	0.8427 - 0.9214	0.736	0.859	0.946	0.491	Train
Combined	0.825	0.889	0.8509 - 0.9276	0.894	0.594	0.881	0.623	Train
Gleason	0.742	0.750	0.6535 - 0.8467					test
Radiomics	0.742	0.732	0.6304 - 0.8343	0.843	0.452	0.815	0.500	test
Habitat	0.758	0.860	0.7925 - 0.9277	0.764	0.742	0.895	0.523	test
Combined	0.825	0.886	0.8183 - 0.9533	0.899	0.613	0.870	0.679	test

**Figure 7:**
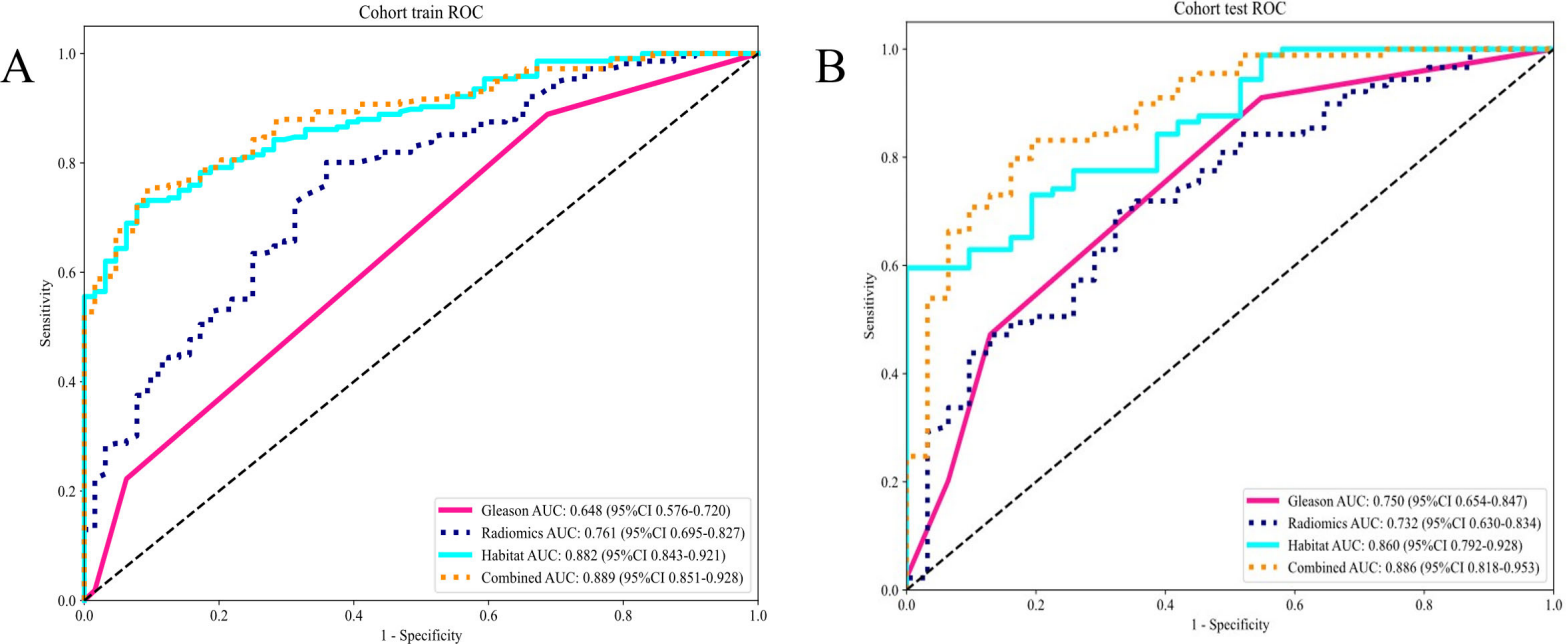
**(A)** Different signatures of area under the receiver operating characteristic (AUROC) curve on the training cohort; **(B)** Different signatures AUROC on the testing cohort.

The findings indicate that the Combined model, harnessing Habitat features and Gleason grading, demonstrates superior predictive accuracy and robustness in both training and testing scenarios. This enhanced performance of the combined model is attributable to the synergistic effect accomplished through the integration of varied and complementary data sources, leading to improved recognition capabilities. The combined model’s consistent capability to achieve higher AUC values across different cohorts highlights its effectiveness and clinical applicability in distinguishing between classes with greater reliability (**Figures 7A, B**).

##### Calibration curve analysis

**Figure 8:**
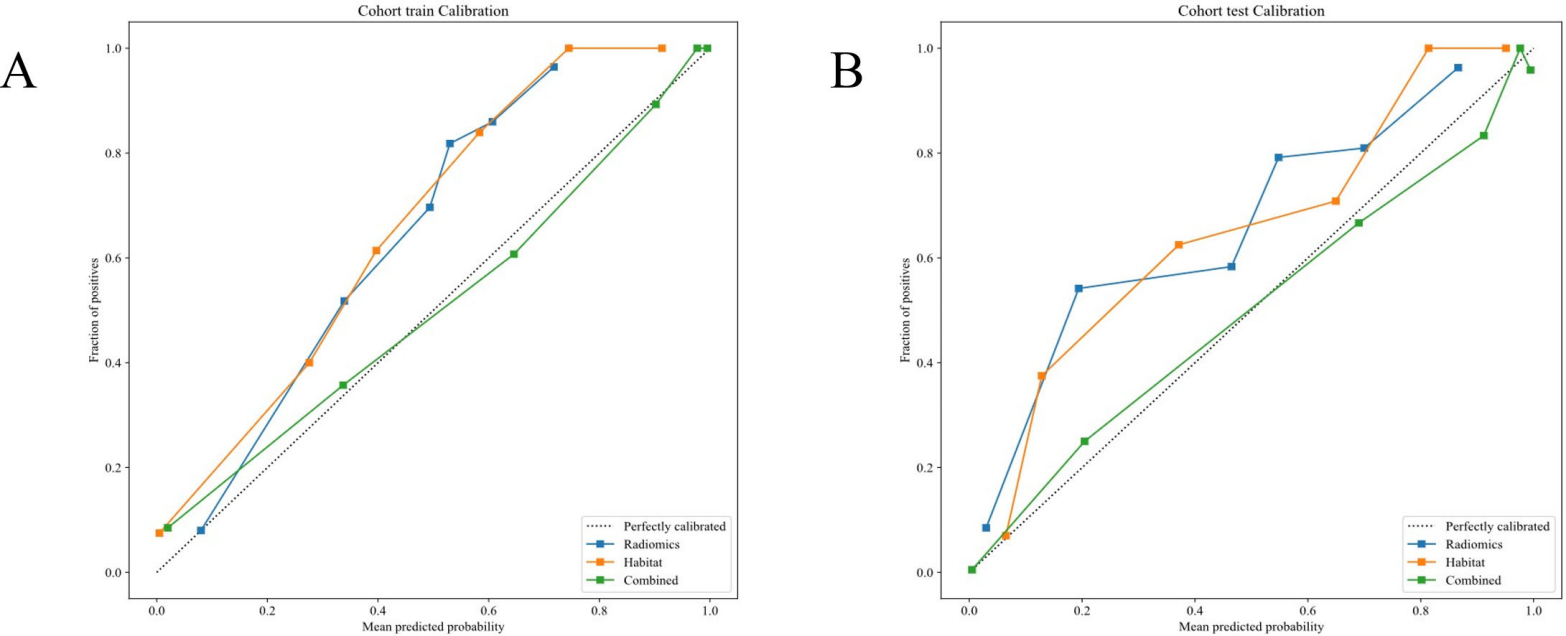
**(A, B)** Different signatures' calibration curves on the testing cohort.

The evaluates model calibration was evaluated by the Hosmer-Lemeshow (HL) test by gauging the consistency between projected probabilities and detected outcomes. Lower HL values suggest better calibration. Excellent calibration was showed by our Nomogram model, with HL values of 0.719 in the training cohort and 0.715 in the testing cohort, indicating high accuracy and reliability in its predictions (**Figures 8A, B**).

##### DeLong test

**Figure 9:**
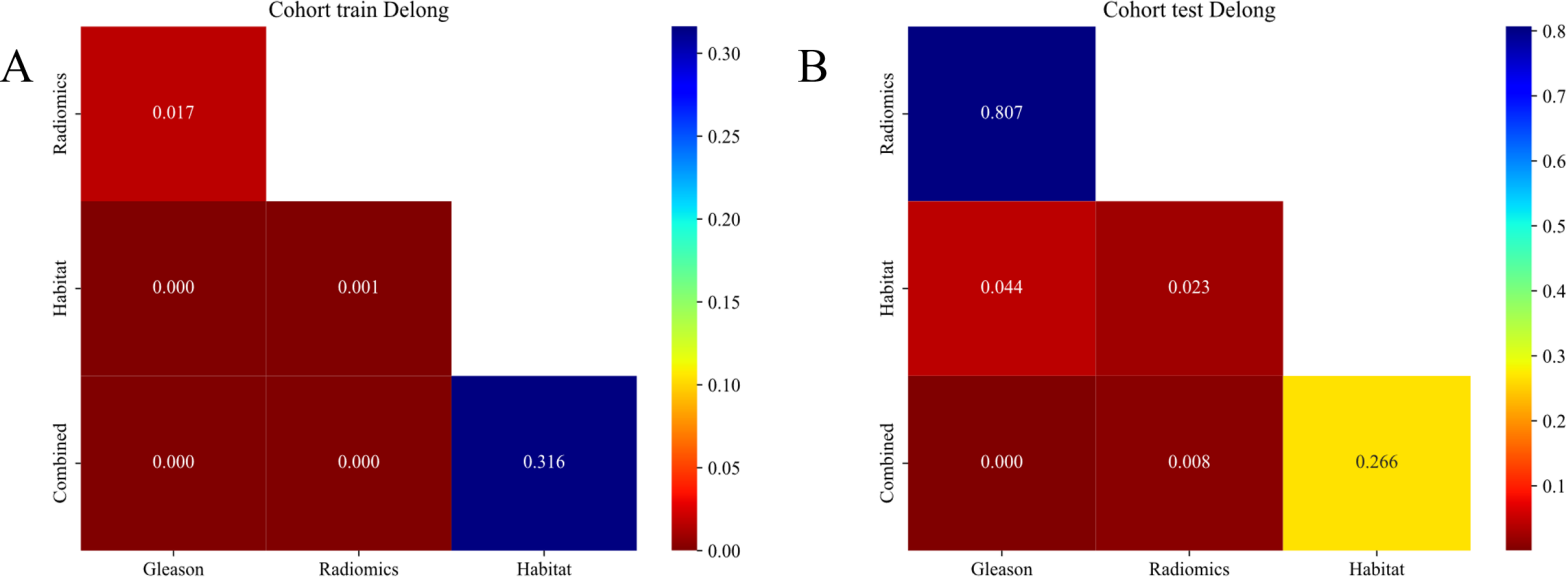
**(A, B)** Delong et al. of **(A)**. cohort and **(B)**. test signatures.

The Habitat model significantly outperformed the Radiomics model in the training cohort as demonstrated by the DeLong test and shows a nearly significant advantage in the testing cohort (22). This superiority suggests that the Habitat model, which focuses on the tumor microenvironment captures the complexities of tumor heterogeneity, more effectively compared to the Radiomics model that primarily employs imaging features. These findings highlight the potential of incorporating tumor microenvironment characteristics to enhance the predictive accuracy of oncological models (**Figures 9A, B**).

#### Clinical use

##### Decision curve analysis

**Figure 10:**
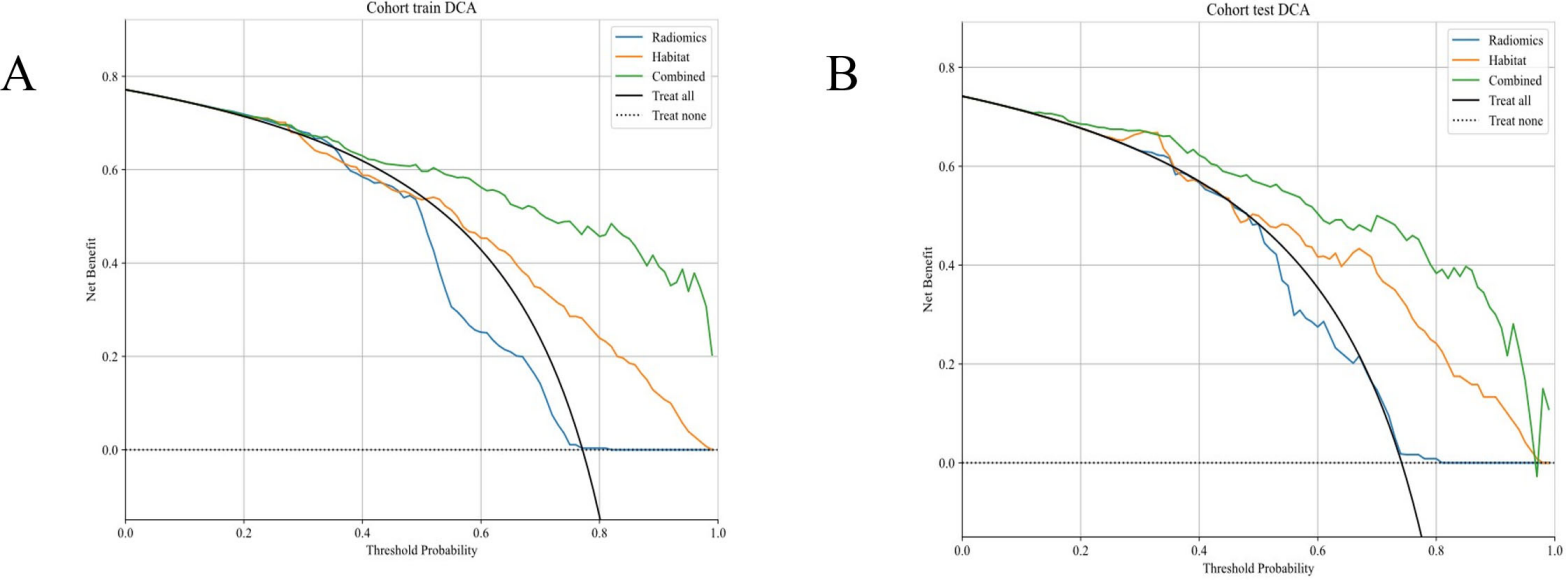
**(A, B)** Different signatures’ decision curves on the testing cohort.

(**Figures 10A, B**) displays the DCA curves for both the training and testing cohorts. The combined model demonstrated a notable advantage in terms of net benefit derived from predicted probabilities.

##### Nomogram

**Figure 11:**
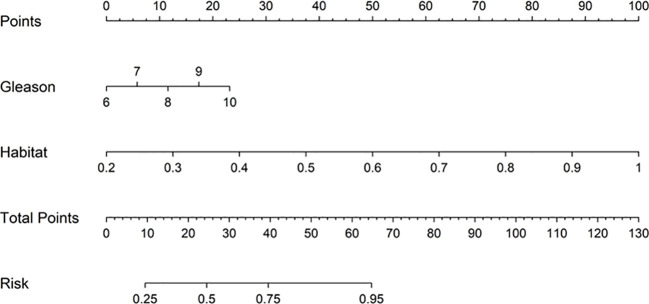
Nomogram constructed based on the integrated model of Habitat and Gleason score.

We employed a Nomogram to visualize the results of our Combined model (**Figure 11**).

## Discussion

PNI is a tropism of cancer cells towards surrounding nerves in the tumor microenvironment. PNI is associated with disease metastasis, recurrence, and poor survival in multiple cancers, including prostate, pancreatic, head and neck, salivary and colon cancers. Usually, cancer metastasis occurs mainly through the lymphatic or vascular conduits, but PNI allows cancer cells to spread along nerve tracts beyond the predicted anatomic borders of a primary tumor. This often results in incomplete surgical resection associated with elevated recurrence rates. Moreover, like many other organs in the body, the prostate is innervated by both sensory and autonomic nerve fibers, creating favorable conditions for cancer cells to spread through the nerves (23) which often leads to adverse outcomes. For example, prostate cancer patients having an invasion of the nerves may have poor recovery of postoperative urinary control and sexual function after undergoing radical prostatectomy, as nerves control urinary control and sexual function, impacting the patient’s quality of life. Therefore, it is necessary to accurately predict the presence of neurological invasion in patients with prostate cancer. Non-invasive examination methods would be more preferable to the patient and would provide more choices for for better treatment planning. After screening a large number of patients in the early stage, we ultimately selected 400 samples that met our requirements for inclusion.

By including appropriate inclusion and exclusion criteria in this study, our team was able to focus on a more defined and homogenous study population, reducing the impact of confounding factors. This helped to enhance the reliability and interpretability of the study results. The patients were divided into two groups, a training group and an internal testing group to ensure the validity and generalizability of the model. Moreover, a new image analysis method was adapted to improve the diagnostic accuracy by intensely analyzing the tumor microenvironment and integrating clinical variables. This method overcomes some of the limitations that are encountered with conventional medical image analysis and provides new ideas for accurate diagnosis and treatment of prostate cancer.

By comprehensively analyzing the tumor microenvironment and radiomics features, we can more deeply reveal the biological characteristics and heterogeneity of prostate cancer. Consequently, the accuracy of diagnosis can be significantly improved, misdiagnosis and missed diagnosis can be effectively reduced, ensuring that patients can receive appropriate treatment in a timely manner. Meanwhile, these features provide valuable evidence for personalized treatment of prostate cancer. Specifically, through a detailed analysis of immune cell infiltration and angiogenesis status in the tumor microenvironment, combined with radiomics features, doctors can formulate more precise treatment plans, which not only improve the treatment effect but also reduce side effects.In addition, the combination of the tumor microenvironment and radiomics models can also accurately predict the responses of prostate cancer patients to different treatment regimens and their prognoses. This ability enables doctors to have a more solid basis for choosing the best treatment plan for patients and to adjust strategies in a timely manner according to the actual situation during the treatment process, thus significantly improving the survival rate and quality of life of patients. More importantly, the analysis results of tumor microenvironment and radiomics features provide strong support for clinical decision-making. For example, when making crucial decisions such as whether to perform radical surgery, radiotherapy, or endocrine therapy, these features have become indispensable important references for doctors.

The analysis of the tumor microenvironment and radiomics features requires close collaboration among multiple disciplines such as radiology, pathology, medical oncology, and surgical oncology. Establishing such a multidisciplinary team can jointly customize more precise diagnosis and treatment plans, significantly improving treatment outcomes. To promote the wide application of this technology in clinical practice, it is essential to provide systematic training for medical staff, covering key skills such as radiomics feature extraction and analysis, tumor microenvironment detection techniques, and the formulation of personalized treatment plans. When integrating these analytical techniques into clinical practice, it is necessary to follow relevant clinical guidelines and consensuses to ensure the rationality and scientific nature of the diagnosis and treatment plans. Meanwhile, continuous monitoring and evaluation of their effectiveness and safety are crucial during the clinical application process. This includes regularly collecting patient clinical data, evaluating treatment efficacy and side effects, so as to timely adjust and optimize treatment plans. In conclusion, the comprehensive analysis of the tumor microenvironment combined with radiomics models has significant clinical implications in the diagnosis and treatment of prostate cancer. Through multidisciplinary collaboration, technical training, establishment of standardized procedures, adherence to clinical guidelines, and continuous monitoring and evaluation, we can effectively integrate this technology into clinical practice and provide patients with more precise and personalized treatment.

An detailed analysis of the tumor microenvironment, which is characterized by a variety of cellular and molecular interactions that can have a significant impact on tumor growth, metastasis, and drug resistance was performed in this study. The characteristics of different tumor regions (24) were comprehensively analyzed, to gain a more complete understanding of the biological characteristics of tumors, and thus more accurately predict disease progression and treatment outcomes of patients (25). Second, by incorporating the patient’s age, non-disease history, and pathologic stage into the model, we could construct a prediction model that was more in line with the actual clinical scenario, furnishing clinicians with more accurate and useful information.

However, our study has several limitations (26). First, though we had a substantial sample size, the cohort size of 400 patients may not be representative of the entire patient population (27). Future studies with larger and more diverse cohorts would facilitate in validating our findings and ensure the generalizability of our approach. Second, the image acquisition and preprocessing protocols, although standardized (28–31), may introduce variability potentally affect the performance of our model. Stricter standardization and quality control measures could help mitigate this issue.

## Conclusion

This study provides new concepts and methods for a more accurate diagnosis and treatment of patients with prostate cancer by introducing an innovative image analysis method to and build a clinical prostate prediction model based on tumor habitat analysis and MRI image histology. Although this study still has some challenges in practical applications, it lays a solid foundation for future medical image analysis and clinical applications.

## Data Availability

The original contributions presented in the study are included in the article/**Supplementary Material**. Further inquiries can be directed to the corresponding authors.
