# Supplementary material for: MRI-based habitat analysis of vascular and nerve invasion in the tumor microenvironment: an advanced approach for prostate cancer diagnosis
Source: Front Oncol. 2025 Apr 17;15:1541413. doi: 10.3389/fonc.2025.1541413 (PMC12043454; doi:10.3389/fonc.2025.1541413)
Supplement: Supplementary file 1 [file DataSheet1.docx]

**Supplementary**

**1A. Intratumor Heterogeneity Generation Process**

Our methodology for delineating tumor habitat regions was multifaceted and involved several complex steps:

1. **Comprehensive Radiomic Feature Extraction:** This process involved extracting detailed local features from each voxel in the dataset using a $3\times3\times3$ moving window. These features encompass a variety of measurements and attributes, including intensity, texture, and other statistical properties, which are crucial for understanding the intricate details of the dataset. Such detailed insights enable more precise modeling and analysis.

- In this study, 19 radiomic features were extracted from each voxel, offering a multidimensional characterization of each subregion. These features included a range of shape descriptors, textural features, and first-order statistical attributes. The specific features extracted were: firstorder_Entropy, firstorder_MeanAbsoluteDeviation, firstorder_Median, glcm_DifferenceAverage, glcm_DifferenceEntropy, glcm_DifferenceVariance, glcm_Imc1, glcm_Imc2, glcm_InverseVariance, glcm_JointEnergy, glcm_JointEntropy, glcm_SumEntropy, glrlm_LongRunEmphasis, glrlm_RunEntropy, glrlm_RunVariance, glszm_SizeZoneNonUniformityNormalized, glszm_SmallAreaHighGrayLevelEmphasis, ngtdm_Contrast, and ngtdm_Strength.
- **Entropy**: Entropy specifies the uncertainty/randomness in the image values.

$$entropy=-\sum_{i=1}^{N_{g}} p\left( i \right)\log_{2}\left( p\left( i \right)+\epsilon\right)$$

- **Mean Absolute Deviation (MAD)**: MAD is the mean distance of all intensity values from the Mean Value of the image array.

$$MAD=\frac{1}{N_{p}}\sum_{i=1}^{N_{p}} \left| X\left( i \right)-X \right|$$

- **Difference Entropy**: Measures the randomness/variability in neighborhood intensity value differences.

$$difference\_entropy=\sum_{k=0}^{N_{g}-1} p_{x-y}\left( k \right)\log_{2}\left( p_{x-y}\left( k \right)+\epsilon\right)$$

- **Difference Variance**: A measure of heterogeneity, giving higher weights to differing intensity level pairs.

$$difference\_variance=\sum_{k=0}^{N_{g}-1} \left( k-DA \right)^{2}p_{x-y}\left( k \right)$$

- **Joint Energy**: A measure of homogeneous patterns in the image.

$$joint\_energy=\sum_{i=1}^{N_{g}} \sum_{j=1}^{N_{g}} \left( p\left( i,j \right) \right)^{2}$$

- **Joint Entropy**: Measures the randomness/variability in neighborhood intensity values.

$$joint\_entropy=-\sum_{i=1}^{N_{g}} \sum_{j=1}^{N_{g}} p\left( i,j \right)\log_{2}\left( p\left( i,j \right)+\epsilon\right)$$

1. **KMeans Subregion Clustering:** The K-means algorithm was employed to analyze the multidimensional feature space derived from the radiomic features. This method clustered all voxels and their associated characteristics, exploring a variety of cluster centers, ranging from 3 to 10, to categorize distinct habitat regions within the tumor. The efficacy of the clustering was assessed using the Calinski-Harabasz score, which facilitated the selection of the most statistically significant clustering configuration.

- The K-means algorithm functions by partitioning data into K distinct clusters. It iteratively updates the centroids of these clusters to minimize the sum of squares within each cluster. The central component of the K-means algorithm is the objective function, which is optimized to achieve effective clustering.

$$J=\sum_{i=1}^{N} \sum_{k=1}^{K} w_{ik}\times\parallel x_{i}-\mu_{k}\parallel^{2}$$

- - $J$ is the objective function.
  - $N$ is the number of data points.
  - $K$ is the number of clusters.
  - $w_{ik}$ is a binary indicator (1 if data point $i$ is in cluster $k$, 0 otherwise).
  - $x_{i}$ is the ith data point.
  - $\mu_{k}$ is the centroid of cluster $k$.
  - $\parallel x_{i}-\mu_{k}\parallel^{2}$ is the squared Euclidean distance between data point $i$ and centroid $k$.

1. **Habitat Region Synthesis:** Following the clustering analysis, subregions with identical cluster IDs were amalgamated. This synthesis resulted in the formation of comprehensive habitat regions, each representing a unique microenvironmental characteristic within the tumor.

**2A. Details of Radiomics Signature**


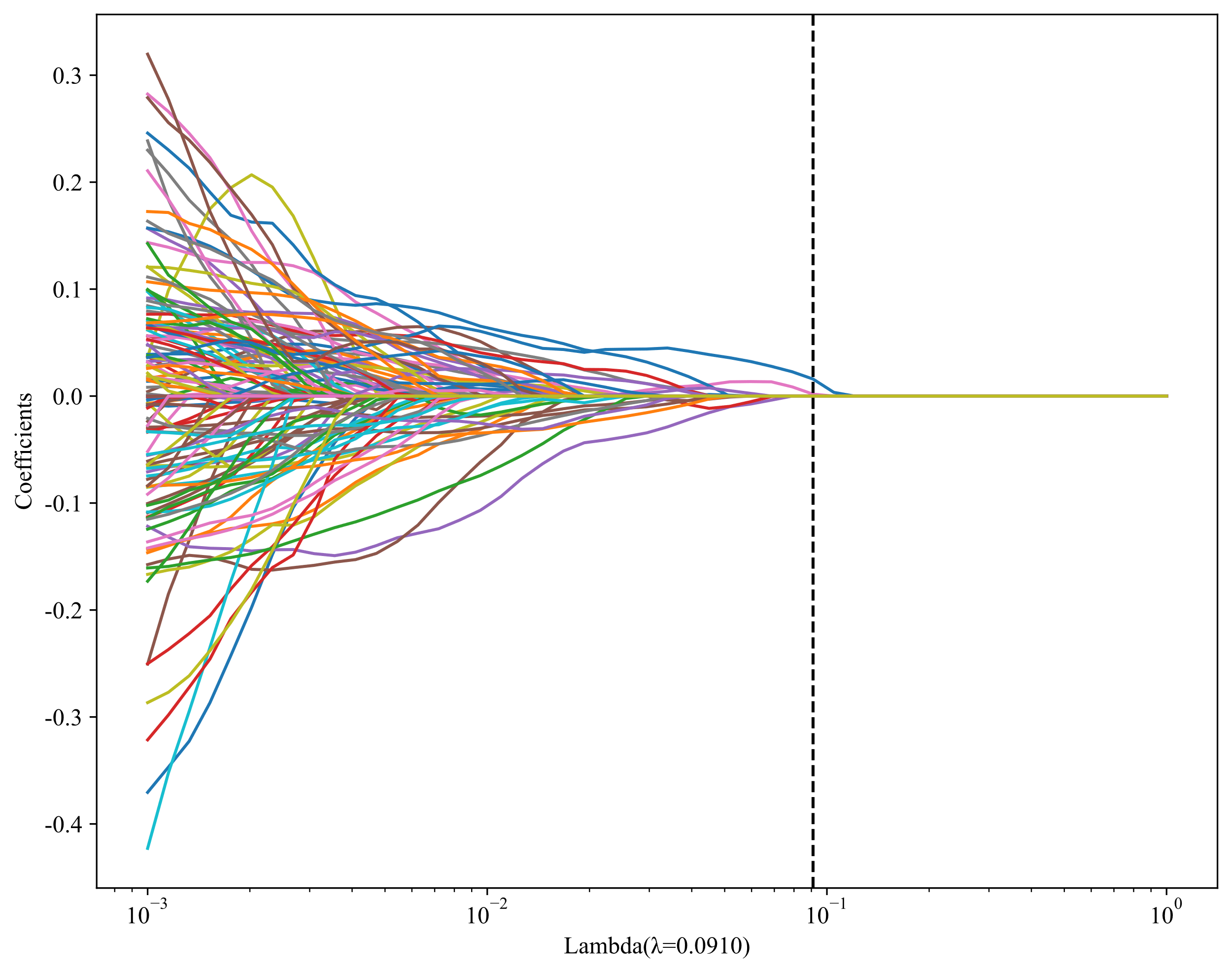

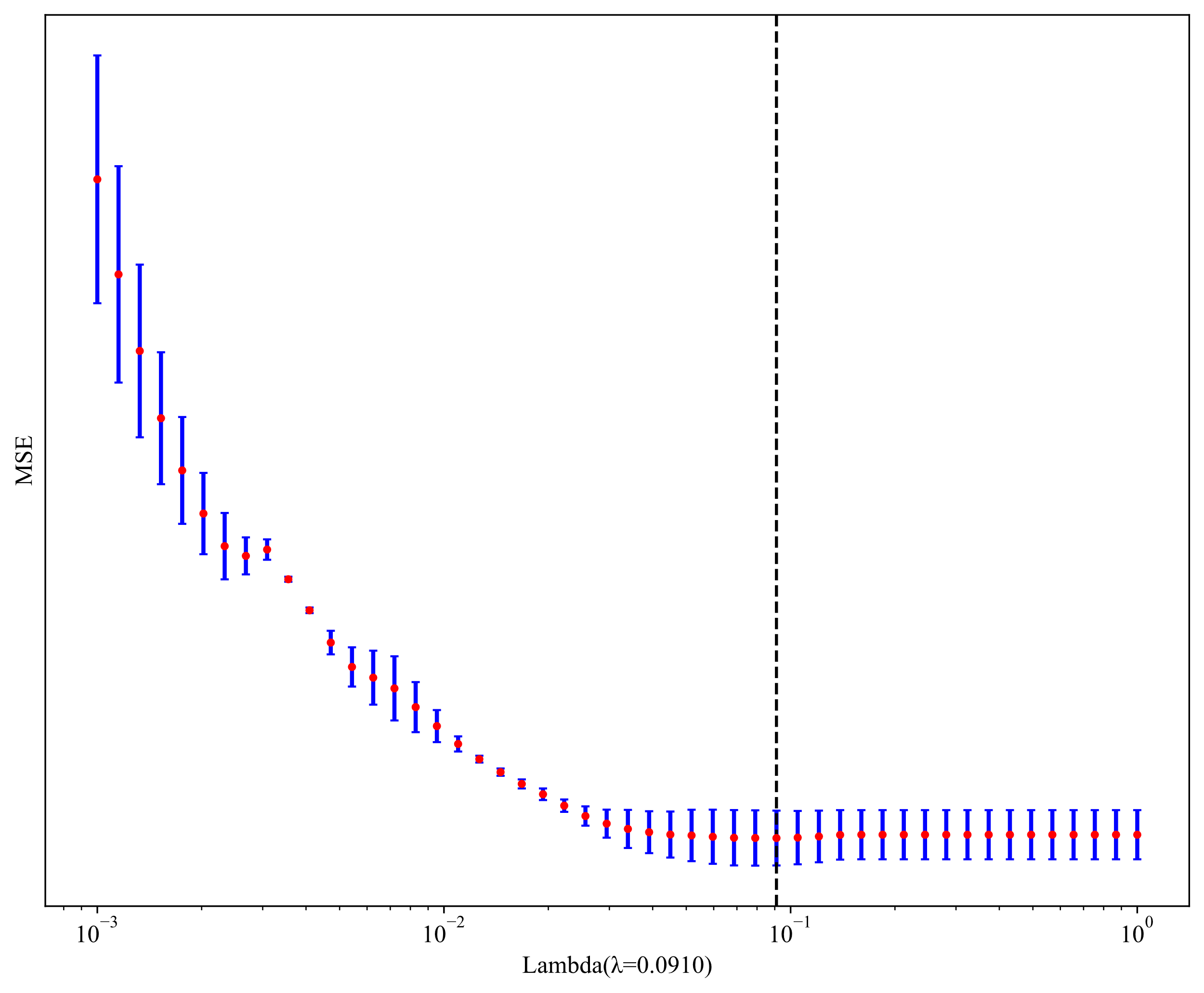

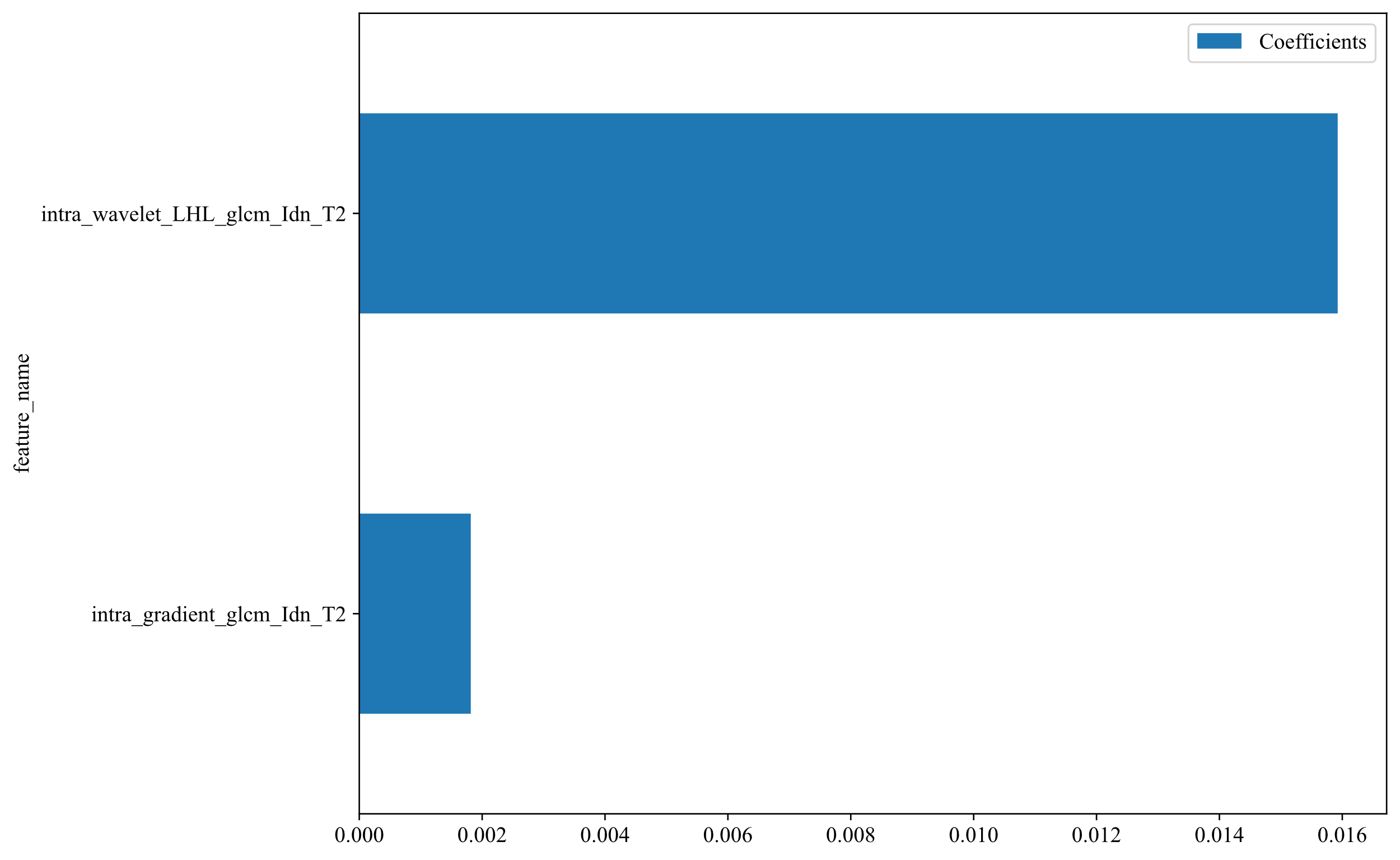


**Supplementary Figure 1:** Coefficients of 10-fold cross validation, Fig.1 MSE of 10 fold cross validation, The histogram of the Rad-score based on the selected features.


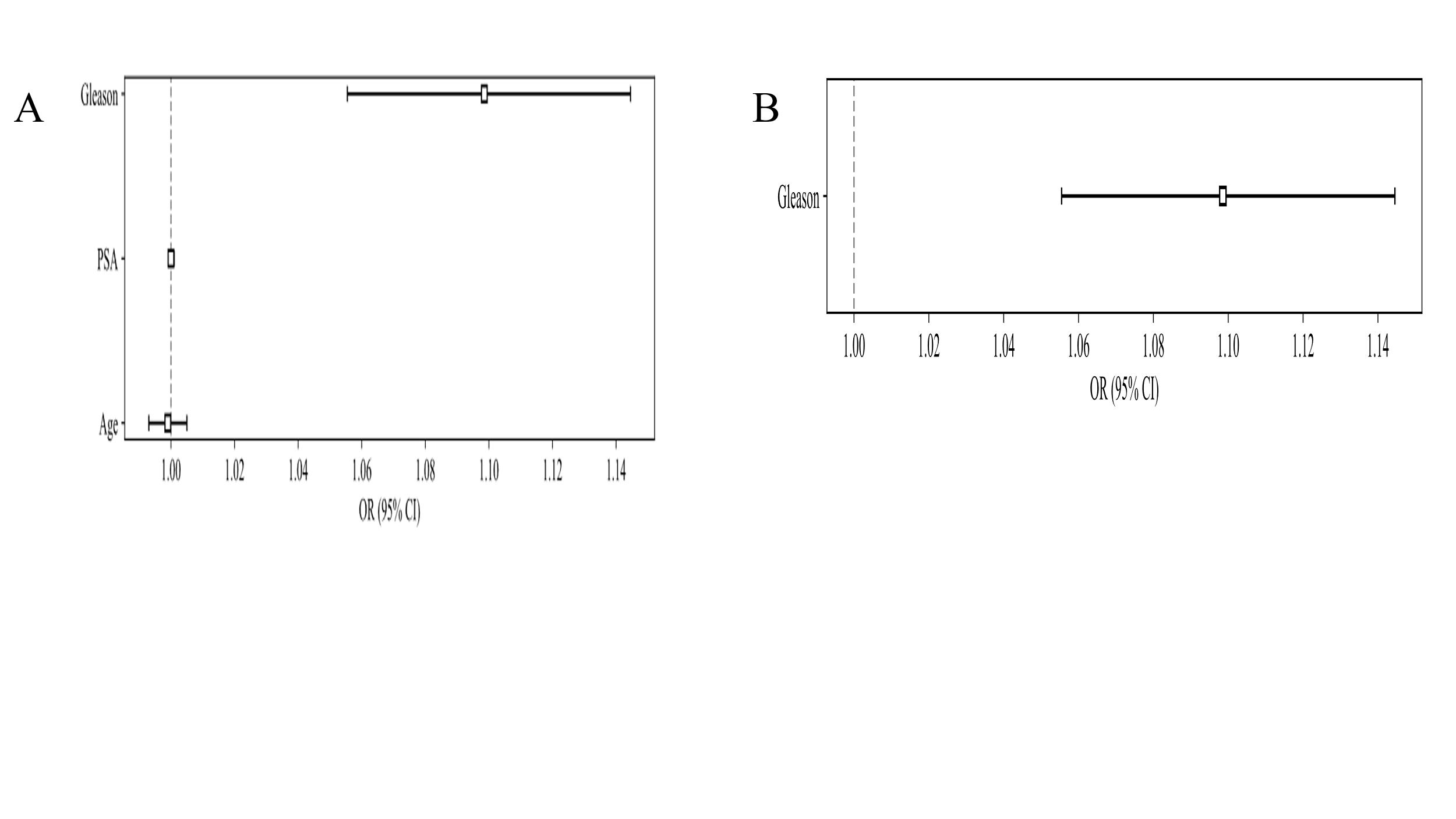


**Supplementary Figure 2: A)** Odds ratio (OR) of clinical features in univariable analysis; **B)** OR of clinical features in multivariable analysis


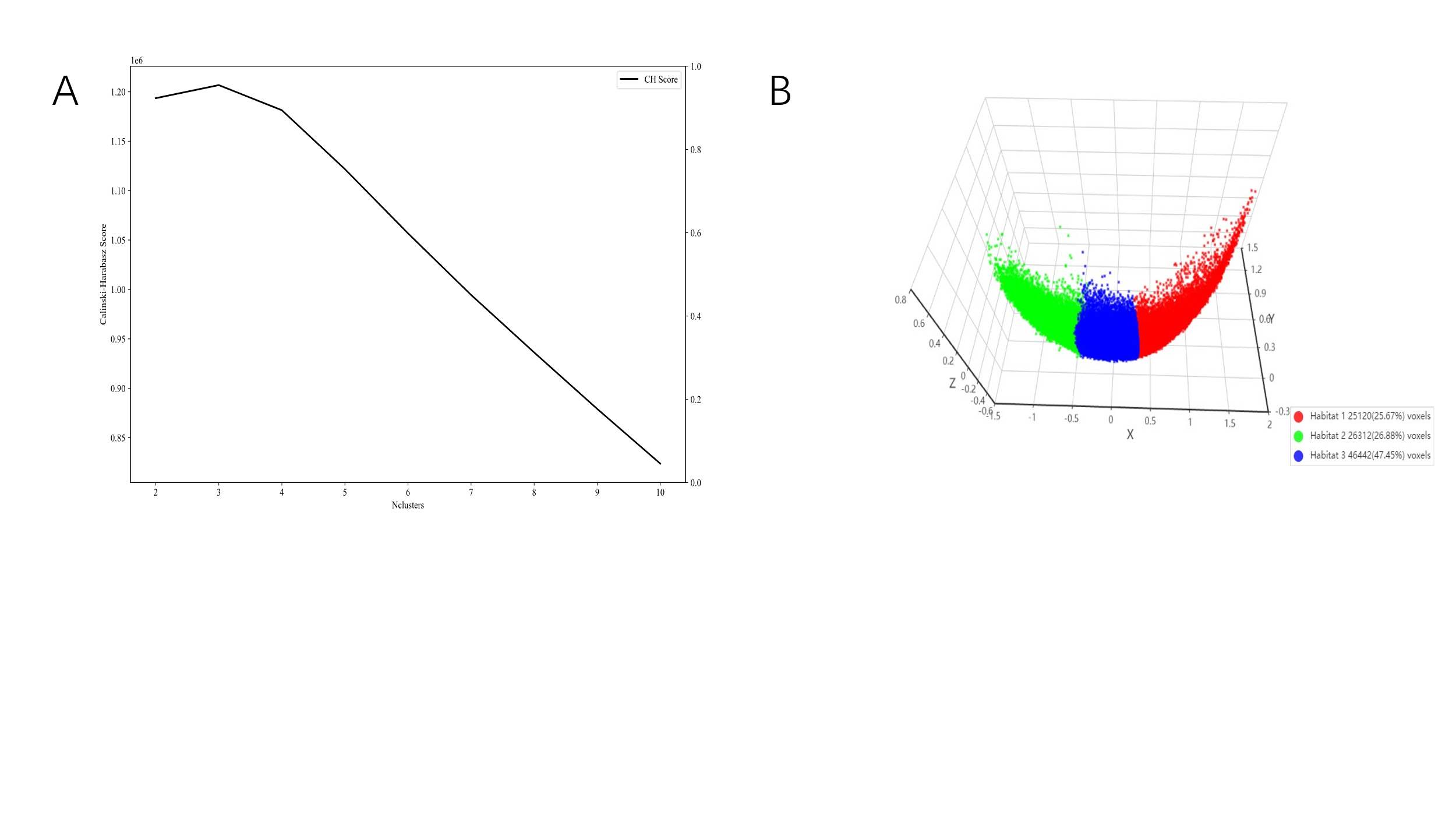


**Supplementary Figure 3: A)** The Calinski-Harabasz (CH) scores for various cluster counts, demonstrating how the number of clusters affects the efficacy of segmentation; **B)** Visualizations of MRI features, segmented into three distinct clusters.

| Sequence | Plane | TR(ms) | TE(ms) | Slice Thickne ss (mm) | Slice Gap (mm) | Fov (mm) | Matrix | B-value (s/mm^2^) |  |
| --- | --- | --- | --- | --- | --- | --- | --- | --- | --- |
| T2WI | Axial | 3000-4000 | 80-120 | 3 | 0.3 | 180-220 | 320*320 | N/A |  |
| T2WI | Sagittal | 3000-4000 | 80-120 | 3 | 0.3 | 180-220 | 320*320 | N/A |  |
| T2WI | Coronal | 3000-4000 | 80-120 | 3 | 0.3 | 180-220 | 320*320 | N/A |  |
| DWI | Axial | 3000-4000 | 60-90 | 3 | 0.3 | 180-220 | 128*128 | 0,50-100,800-1000 |  |
| DCE | Axial | 4-6 | 2-3 | 3 | 0 | 180-220 | 256*256 | N/A |  |

**Supplementary Table 1**: Various parameters of 3.0 Tesla Philips MRI machine
